# 10.3 GLIA-EXTRACELLULAR MATRIX INTERACTIONS IN THE PATHOPHYSIOLOGY OF SCHIZOPHRENIA AND BIPOLAR DISORDER

**DOI:** 10.1093/schbul/sby014.036

**Published:** 2018-04-01

**Authors:** Sabina Berretta, Gabriele Chelini, Harry Pantazopoulos

**Affiliations:** Harvard Medical School, McLean Hospital

## Abstract

**Background:**

Growing evidence from our group and others indicates that key neural functions, including regulation of synaptic plasticity and axonal guidance and connectivity, arise from interactions between glial cells, neurons, and the extracellular matrix. Several distinct populations of glial cells critically contribute to the composition of main components of the extracellular matrix (ECM), synthesizing them and secreting them into the extracellular space, where they become incorporated in organized ECM structures. The brain ECM, and chondroitin sulfate proteoglycans (CSPGs) in particular, play a key role in brain development and adult life, in turn regulating glial functions as well as synaptic plasticity and neural connectivity. We have previously shown that glial cells expressing CSPGs are altered in the amygdala and entorhinal cortex of people with schizophrenia (SZ) and bipolar disorder (BD). These changes are accompanied by marked decreases of perineuronal nets (PNNs), organized ECM structures unsheathing distinct neuronal populations. Recent and ongoing studies are focused on novel CSPG-enriched ECM structures, related to synaptic complexes and myelinated axons, their relationship to glial populations and their involvement in the pathophysiology of SZ and BD.

**Methods:**

Postmortem tissue samples from the amygdala, entorhinal cortex and thalamus from a well characterized cohort of healthy control, SZ and BD subjects were included in these studies. Multiplex immunofluorescence combined with quantitative microscopy was used to quantify glial cells and CSPGs, while electron microscopy on human and mouse tissue were used to investigate ultrastructural morphology. Step-wise ANOVA analyses included several potential confounds such as exposure to pharmacological agents and substance abuse.

**Results:**

Our results show that at least two novel ECM structures are present in the human brain. The first, enriched in CSPGs bearing chondroitin sulfation in position 6 (CS-6), and named here ‘CS-6 clusters’ was found to be markedly decreased in the amygdala of people with SZ and BD. Electron microscopy studies show that CS-6 clusters are composed of astrocytes synthesizing and secreting CS-6 CSPGs in the vicinity of adjacent groups of dendrites, where it is incorporated into postsynaptic densities of dendritic spines. The second CSPG-enriched ECM structure, i.e. axonal coats, has been observed in the human thalamus to envelope distinct populations of axons, interweaving with myelin sheets. Its main CSPG components appear to be synthesized and secreted by oligodendrocytes precursor cells located in the vicinity of axon bundles. Preliminary results show abnormalities affecting both oligodendrocyte precursors and axonal coats in SZ.

**Discussion:**

In summary, our results show complex interactions between glial cells, neurons and ECM, potentially affecting synaptic functions and axonal conductance. Results in SZ and BD point to a profound disruption of these interactions in several brain regions.

